# Vascular irregularities in COVID-19: the relevance of computed tomography pulmonary angiography in the diagnosis of vascular complications

**DOI:** 10.1590/0100-3984.2024.57.e10-en

**Published:** 2024-11-29

**Authors:** Alexandre Dias Mançano

**Affiliations:** 1 PhD in Radiology from the Universidade Federal do Rio de Janeiro (UFRJ), Rio de Janeiro, RJ, Brazil, Thoracic Radiologist at Hospital Sírio-Libanês Brasília, Brasília, DF, Brazil. Email:alex.manzano1@gmail.com.

Since the beginning of the coronavirus disease 2019 (COVID-19) pandemic, the systemic manifestations of infection with severe acute respiratory syndrome coronavirus 2 (SARS-CoV-2) have been extensively investigated. In addition to the widely known respiratory complications, robust evidence of vascular repercussions related to the disease is emerging, consolidating the understanding of COVID-19 as a disease that has severe, systemic effects on the vascular system, the detection of which has increasingly been the object of investigation^(1-5)^.

The study conducted by Nobre et al.^(6)^ and published in Radiologia Brasileira offers valuable contributions by addressing the observation of vascular wall irregularities on computed tomography pulmonary angiography (CTPA) examinations of patients with COVID-19 pneumonia. The authors retrospectively analyzed the CTPAs of 65 patients diagnosed with COVID-19 and observed that the vessel wall irregularities sign was present in 76.9% of cases, predominantly in subsegmental pulmonary arteries and veins, most commonly in the lower lobes, being bilateral in 92% of the patients. These findings confirm that COVID-19 is a diffuse disease with extensive involvement of the pulmonary circulatory system, rather than a disease confined exclusively to the respiratory system, thus supporting the role of CTPA as a crucial tool in the diagnosis of vascular complications related to COVID-19^(4,5)^.

The detailed analysis of vascular irregularities detected by CTPA in patients with COVID-19 in the Nobre et al.^(6)^ study may reflect the prothrombotic state exacerbated by SARS-CoV-2 infection in these patients, supporting the hypothesis that SARSCoV-2-induced microvascular impairment plays a central role in the pathogenesis of COVID-19-related thrombotic complications and severity.

The identification of vessel wall irregularities on CTPA provides an additional relevant parameter for clinical risk stratification in patients with COVID-19, suggesting that the finding is strongly associated with unfavorable outcomes, including a higher incidence of deep vein thrombosis, pulmonary embolism, and multiple organ failure^(2,7)^. Therefore, CTPA not only represents an essential diagnostic tool, but also guides therapeutic decision-making, especially with regard to the early institution of anticoagulation in patients affected by the disease. In addition, early detection of vascular alterations by CTPA can directly influence the management of COVID-19, especially in patients with less significant respiratory symptoms, allowing intervention at earlier stages of the disease, as well as preventing progression to severe forms and the occurrence of potentially fatal thrombotic complications^(8)^. Therefore, CTPA plays a central role not only in the initial diagnosis but also in the continuous monitoring and personalized treatment of patients affected by the disease.

The contribution that the Nobre et al.^(6)^ study makes to the scientific and medical literature is undeniable, particularly in the context of COVID-19, in which the identification of vascular complications has been a topic of increasing interest and importance. The vascular wall irregularities sign, described in detail in this work, adds a new dimension to the understanding of the manifestations of COVID-19 on CTPA, offering radiologists, pulmonologists, intensivists, and infectious disease specialists an additional clinical indicator that can be used to improve diagnostic accuracy and guide therapeutic approaches, with the objective of reducing the rates of complications and mortality associated with the disease.

As knowledge about COVID-19 advances, it is crucial that additional studies be conducted to elucidate the mechanisms by which infection with SARS-CoV-2 affects the vascular system so extensively and severely. Such knowledge could result in more effective therapeutic strategies with the potential to improve clinical outcomes in patients affected by the disease^(1)^. In this context, dual-energy CT has been used to assess lung perfusion abnormalities in patients with COVID-19. Idilman et al.^(9)^ found lung perfusion deficits in 25.8% of patients and found those deficits to be associated with greater disease severity, as well as with elevated levels of inflammatory and coagulation markers. Mohamed et al.^(10)^ also reported perfusion abnormalities in 87.4% of patients with post-acute COVID-19 syndrome, suggesting that microangiopathy and hypercoagulability persist after the acute phase of the disease.

The study published in Radiologia Brasileira not only expands the understanding of vascular complications associated with COVID-19 but also reaffirms the relevance of CTPA as an indispensable tool in the clinical context of the pandemic. The ongoing, judicious use of this diagnostic technique can undoubtedly contribute to the early detection of complications, providing timely therapeutic interventions, helping to save lives in a challenging, dynamic clinical scenario.
